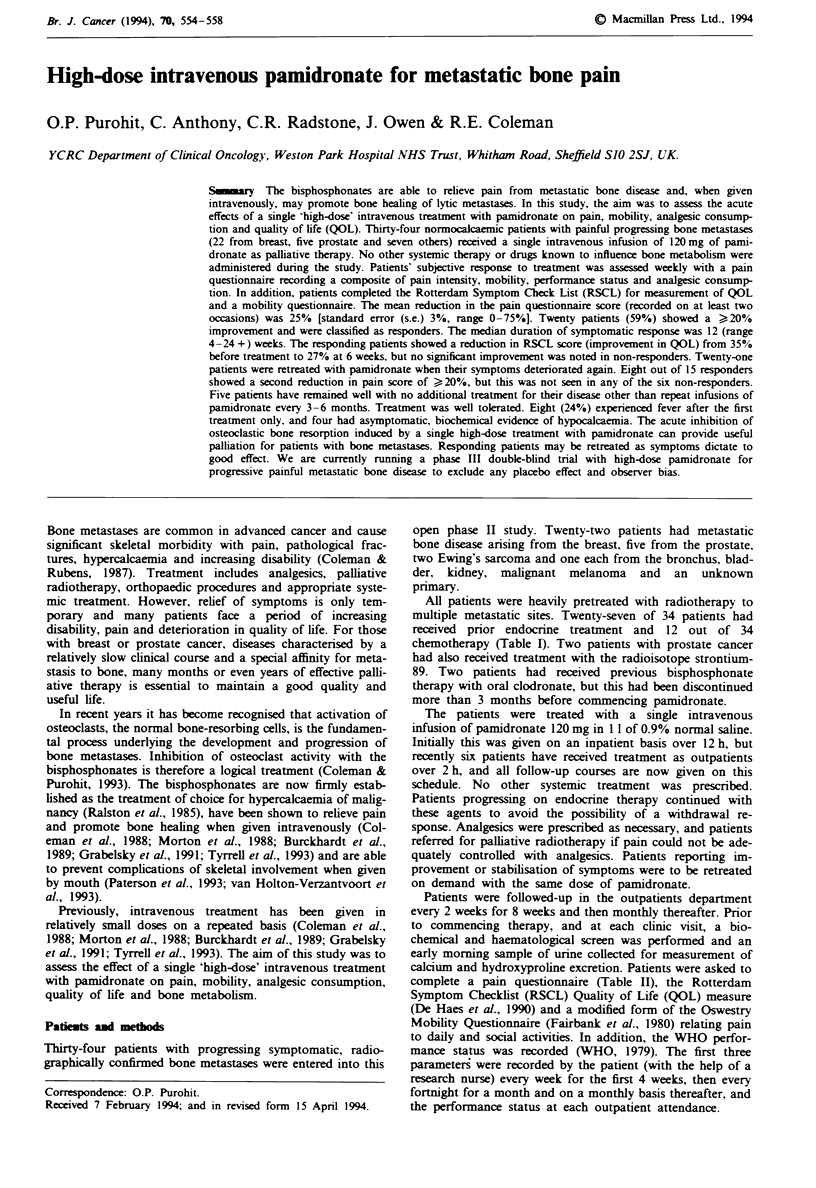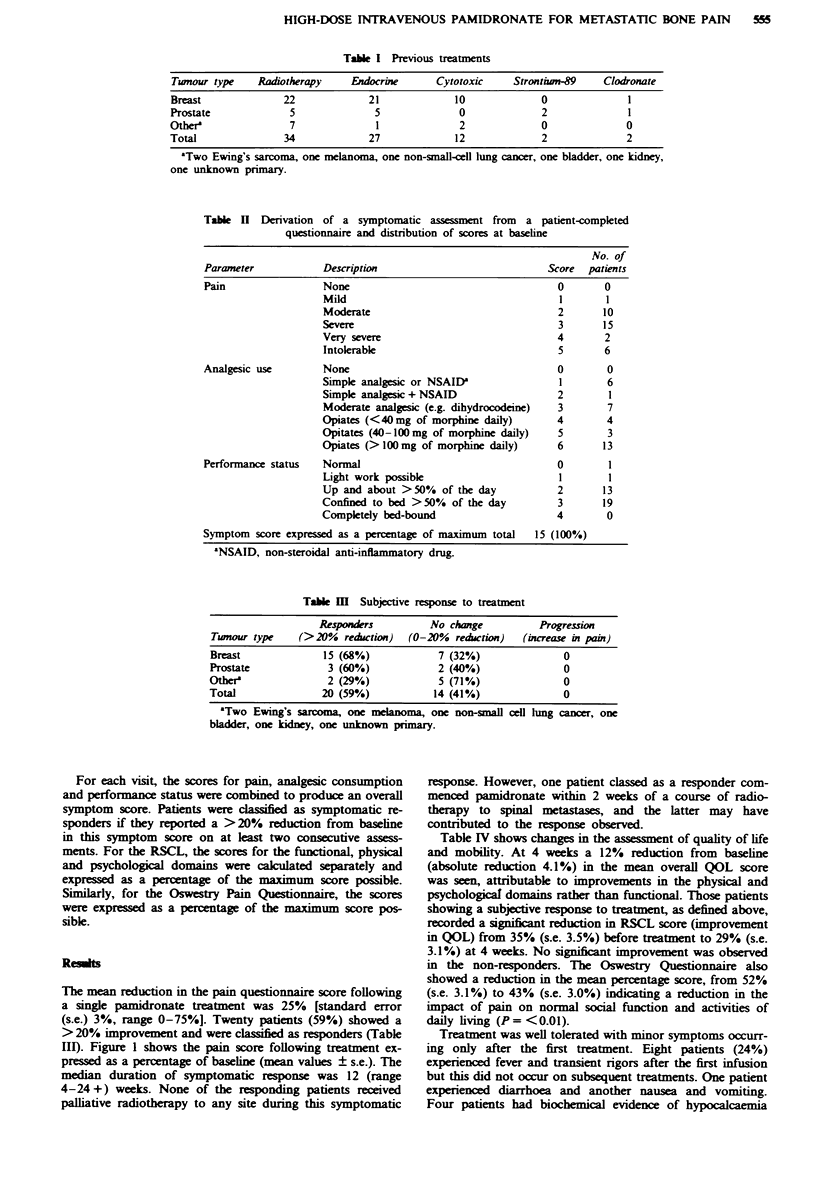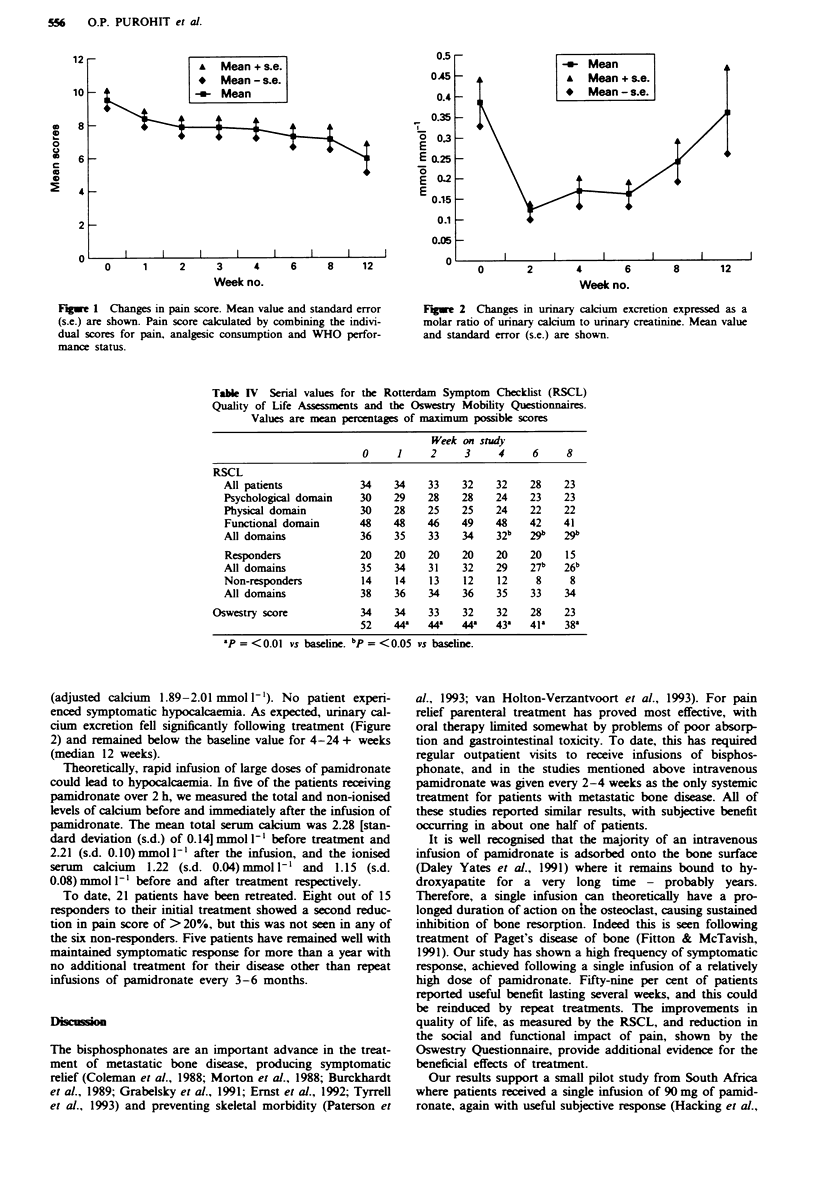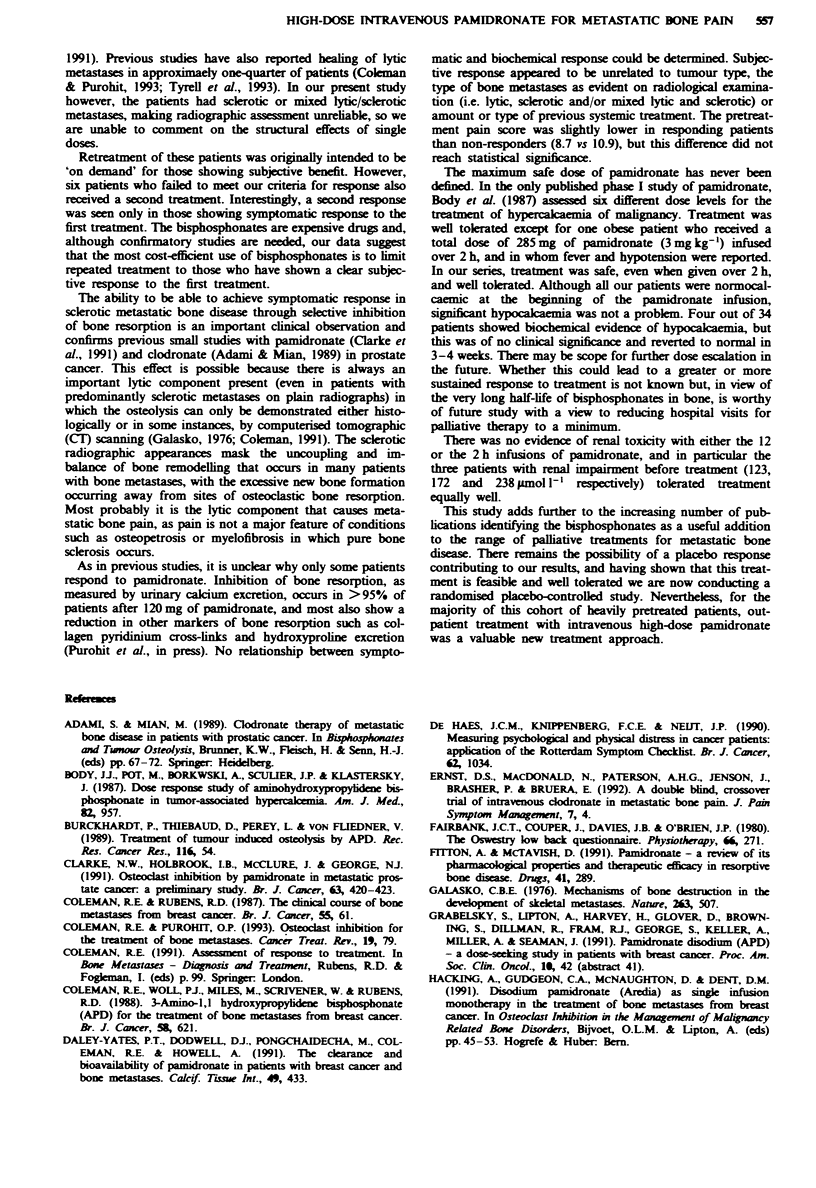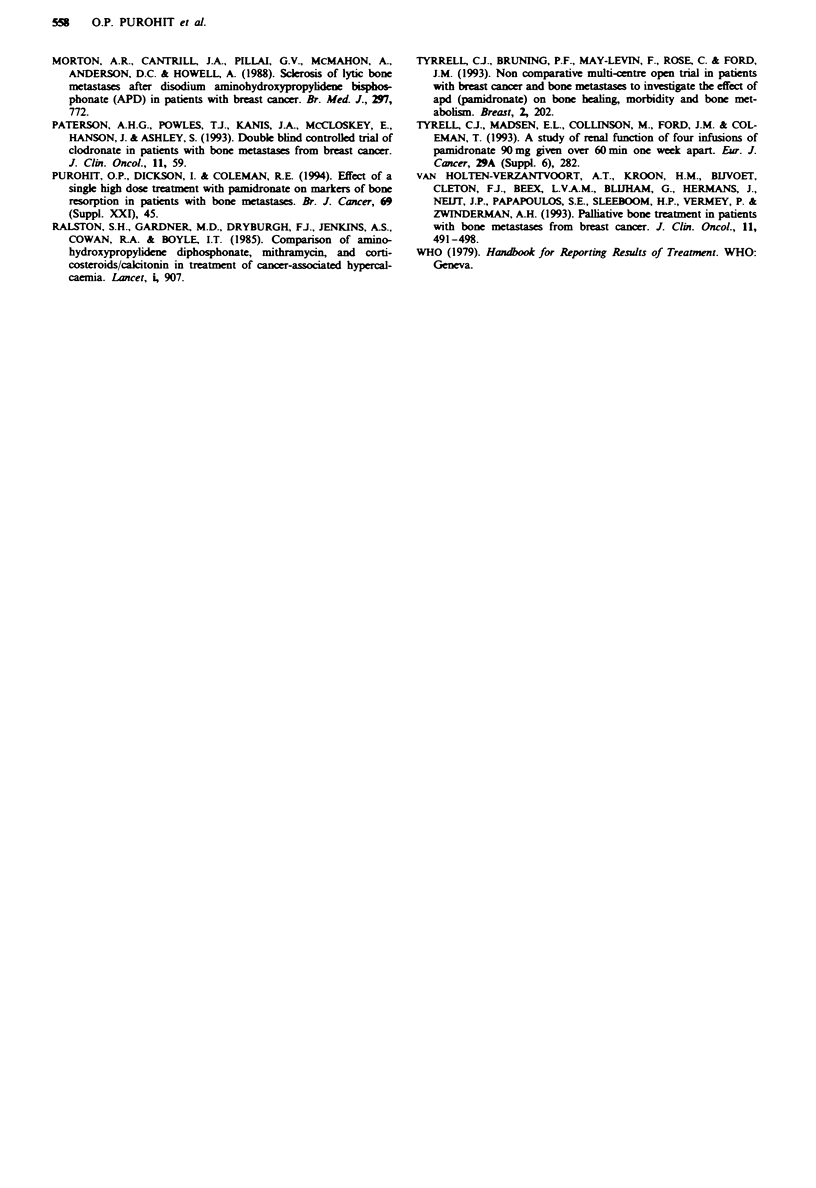# High-dose intravenous pamidronate for metastatic bone pain.

**DOI:** 10.1038/bjc.1994.344

**Published:** 1994-09

**Authors:** O. P. Purohit, C. Anthony, C. R. Radstone, J. Owen, R. E. Coleman

**Affiliations:** YCRC Department of Clinical Oncology, Weston Park Hospital NHS Trust, Sheffield, UK.

## Abstract

The bisphosphonates are able to relieve pain from metastatic bone disease and, when given intravenously, may promote bone healing of lytic metastases. In this study, the aim was to assess the acute effects of a single 'high-dose' intravenous treatment with pamidronate on pain, mobility, analgesic consumption and quality of life (QOL). Thirty-four normocalcaemic patients with painful progressing bone metastases (22 from breast, five prostate and seven others) received a single intravenous infusion of 120 mg of pamidronate as palliative therapy. No other systemic therapy or drugs known to influence bone metabolism were administered during the study. Patients' subjective response to treatment was assessed weekly with a pain questionnaire recording a composite of pain intensity, mobility, performance status and analgesic consumption. In addition, patients completed the Rotterdam Symptom Check List (RSCL) for measurement of QOL and a mobility questionnaire. The mean reduction in the pain questionnaire score (recorded on at least two occasions) was 25% [standard error (s.e.) 3%, range 0-75%]. Twenty patients (59%) showed a > or = 20% improvement and were classified as responders. The median duration of symptomatic response was 12 (range 4-24 +) weeks. The responding patients showed a reduction in RSCL score (improvement in QOL) from 35% before treatment to 27% at 6 weeks, but no significant improvement was noted in non-responders. Twenty-one patients were retreated with pamidronate when their symptoms deteriorated again. Eight out of 15 responders showed a second reduction in pain score of > or = 20%, but this was not seen in any of the six non-responders. Five patients have remained well with no additional treatment for their disease other than repeat infusions of pamidronate every 3-6 months. Treatment was well tolerated. Eight (24%) experienced fever after the first treatment only, and four had asymptomatic, biochemical evidence of hypocalcaemia. The acute inhibition of osteoclastic bone resorption induced by a single high-dose treatment with pamidronate can provide useful palliation for patients with bone metastases. Responding patients may be retreated as symptoms dictate to good effect. We are currently running a phase III double-blind trial with high-dose pamidronate for progressive painful metastatic bone disease to exclude any placebo effect and observer bias.


Br. J. Cancer (1994), 70, 554-558 © Macmillan Press Ltd., 1994

### High-dose intravenous pamidronate for metastatic bone pain

O.P. Purohit, C. Anthony, C.R. Radstone, J. Owen & R.E. Coleman

*YCRC Department of Clinical Oncology, Weston Park Hospital NHS Trust, Whitham Road, Sheffield S10 2SJ, UK.*

**Summary** The bisphosphonates are able to relieve pain from metastatic bone disease and, when given intravenously, may promote bone healing of lytic metastases. In this study, the aim was to assess the acute effects of a single 'high-dose' intravenous treatment with pamidronate on pain, mobility, analgesic consumption and quality of life (QOL). Thirty-four normocalcaemic patients with painful progressing bone metastases (22 from breast, five prostate and seven others) received a single intravenous infusion of 120 mg of pamidronate as palliative therapy. No other systemic therapy or drugs known to influence bone metabolism were administered during the study. Patients' subjective response to treatment was assessed weekly with a pain questionnaire recording a composite of pain intensity, mobility, performance status and analgesic consumption. In addition, patients completed the Rotterdam Symptom Check List (RSCL) for measurement of QOL and a mobility questionnaire. The mean reduction in the pain questionnaire score (recorded on at least two occasions) was 25% [standard error (s.e.) 3%, range 0-75%]. Twenty patients (59%) showed a ≥20% improvement and were classified as responders. The median duration of symptomatic response was 12 (range 4-24+) weeks. The responding patients showed a reduction in RSCL score (improvement in QOL) from 35% before treatment to 27% at 6 weeks, but no significant improvement was noted in non-responders. Twenty-one patients were retreated with pamidronate when their symptoms deteriorated again. Eight out of 15 responders showed a second reduction in pain score of ≥20%, but this was not seen in any of the six non-responders. Five patients have remained well with no additional treatment for their disease other than repeat infusions of pamidronate every 3-6 months. Treatment was well tolerated. Eight (24%) experienced fever after the first treatment only, and four had asymptomatic, biochemical evidence of hypocalcaemia. The acute inhibition of osteoclastic bone resorption induced by a single high-dose treatment with pamidronate can provide useful palliation for patients with bone metastases. Responding patients may be retreated as symptoms dictate to good effect. We are currently running a phase III double-blind trial with high-dose pamidronate for progressive painful metastatic bone disease to exclude any placebo effect and observer bias.

Bone metastases are common in advanced cancer and cause significant skeletal morbidity with pain, pathological fractures, hypercalcaemia and increasing disability (Coleman & Rubens, 1987). Treatment includes analgesics, palliative radiotherapy, orthopaedic procedures and appropriate systemic treatment. However, relief of symptoms is only temporary and many patients face a period of increasing disability, pain and deterioration in quality of life. For those with breast or prostate cancer, diseases characterised by a relatively slow clinical course and a special affinity for metastasis to bone, many months or even years of effective palliative therapy is essential to maintain a good quality and useful life.

In recent years it has become recognised that activation of osteoclasts, the normal bone-resorbing cells, is the fundamental process underlying the development and progression of bone metastases. Inhibition of osteoclast activity with the bisphosphonates is therefore a logical treatment (Coleman & Purohit, 1993). The bisphosphonates are now firmly established as the treatment of choice for hypercalcaemia of malignancy (Ralston *et al.*, 1985), have been shown to relieve pain and promote bone healing when given intravenously (Coleman *et al.*, 1988; Morton *et al.*, 1988; Burckhardt *et al.*, 1989; Grabelsky *et al.*, 1991; Tyrrell *et al.*, 1993) and are able to prevent complications of skeletal involvement when given by mouth (Paterson *et al.*, 1993; van Holton-Verzantvoort *et al.*, 1993).

Previously, intravenous treatment has been given in relatively small doses on a repeated basis (Coleman *et al.*, 1988; Morton *et al.*, 1988; Burckhardt *et al.*, 1989; Grabelsky *et al.*, 1991; Tyrrell *et al.*, 1993). The aim of this study was to assess the effect of a single 'high-dose' intravenous treatment with pamidronate on pain, mobility, analgesic consumption, quality of life and bone metabolism.

### Patients and methods

Thirty-four patients with progressing symptomatic, radiographically confirmed bone metastases were entered into this open phase II study. Twenty-two patients had metastatic bone disease arising from the breast, five from the prostate, two Ewing's sarcoma and one each from the bronchus, bladder, kidney, malignant melanoma and an unknown primary.

All patients were heavily pretreated with radiotherapy to multiple metastatic sites. Twenty-seven of 34 patients had received prior endocrine treatment and 12 out of 34 chemotherapy (Table I). Two patients with prostate cancer had also received treatment with the radioisotope strontium-89. Two patients had received previous bisphosphonate therapy with oral clodronate, but this had been discontinued more than 3 months before commencing pamidronate.

The patients were treated with a single intravenous infusion of pamidronate 120 mg in 1 l of 0.9% normal saline. Initially this was given on an inpatient basis over 12 h, but recently six patients have received treatment as outpatients over 2 h, and all follow-up courses are now given on this schedule. No other systemic treatment was prescribed. Patients progressing on endocrine therapy continued with these agents to avoid the possibility of a withdrawal response. Analgesics were prescribed as necessary, and patients referred for palliative radiotherapy if pain could not be adequately controlled with analgesics. Patients reporting improvement or stabilisation of symptoms were to be retreated on demand with the same dose of pamidronate.

Patients were followed-up in the outpatients department every 2 weeks for 8 weeks and then monthly thereafter. Prior to commencing therapy, and at each clinic visit, a biochemical and haematological screen was performed and an early morning sample of urine collected for measurement of calcium and hydroxyproline excretion. Patients were asked to complete a pain questionnaire (Table II), the Rotterdam Symptom Checklist (RSCL) Quality of Life (QOL) measure (De Haes *et al.*, 1990) and a modified form of the Oswestry Mobility Questionnaire (Fairbank *et al.*, 1980) relating pain to daily and social activities. In addition, the WHO performance status was recorded (WHO, 1979). The first three parameters were recorded by the patient (with the help of a research nurse) every week for the first 4 weeks, then every fortnight for a month and on a monthly basis thereafter, and the performance status at each outpatient attendance.

Correspondence: O.P. Purohit.
Received 7 February 1994; and in revised form 15 April 1994.

**Table I** Previous treatments

| *Tumour type* | *Radiotherapy* | *Endocrine* | *Cytotoxic* | *Strontium-89* | *Clodronate* |
|---|---|---|---|---|---|
| Breast | 22 | 21 | 10 | 0 | 1 |
| Prostate | 5 | 5 | 0 | 2 | 1 |
| Other[a] | 7 | 1 | 2 | 0 | 0 |
| Total | 34 | 27 | 12 | 2 | 2 |

[a]Two Ewing's sarcoma, one melanoma, one non-small-cell lung cancer, one bladder, one kidney, one unknown primary.

**Table II** Derivation of a symptomatic assessment from a patient-completed questionnaire and distribution of scores at baseline

| *Parameter* | *Description* | *Score* | *No. of patients* |
|---|---|---|---|
| Pain | None | 0 | 0 |
| | Mild | 1 | 1 |
| | Moderate | 2 | 10 |
| | Severe | 3 | 15 |
| | Very severe | 4 | 2 |
| | Intolerable | 5 | 6 |
| Analgesic use | None | 0 | 0 |
| | Simple analgesic or NSAID[a] | 1 | 6 |
| | Simple analgesic + NSAID | 2 | 1 |
| | Moderate analgesic (e.g. dihydrocodeine) | 3 | 7 |
| | Opiates (<40 mg of morphine daily) | 4 | 4 |
| | Opitates (40-100 mg of morphine daily) | 5 | 3 |
| | Opiates (>100 mg of morphine daily) | 6 | 13 |
| Performance status | Normal | 0 | 1 |
| | Light work possible | 1 | 1 |
| | Up and about >50% of the day | 2 | 13 |
| | Confined to bed >50% of the day | 3 | 19 |
| | Completely bed-bound | 4 | 0 |
| Symptom score expressed as a percentage of maximum total | | 15 (100%) | |

[a]NSAID, non-steroidal anti-inflammatory drug.

**Table III** Subjective response to treatment

| *Tumour type* | *Responders (>20% reduction)* | *No change (0-20% reduction)* | *Progression (increase in pain)* |
|---|---|---|---|
| Breast | 15 (68%) | 7 (32%) | 0 |
| Prostate | 3 (60%) | 2 (40%) | 0 |
| Other[a] | 2 (29%) | 5 (71%) | 0 |
| Total | 20 (59%) | 14 (41%) | 0 |

[a]Two Ewing's sarcoma, one melanoma, one non-small cell lung cancer, one bladder, one kidney, one unknown primary.

For each visit, the scores for pain, analgesic consumption and performance status were combined to produce an overall symptom score. Patients were classified as symptomatic responders if they reported a >20% reduction from baseline in this symptom score on at least two consecutive assessments. For the RSCL, the scores for the functional, physical and psychological domains were calculated separately and expressed as a percentage of the maximum score possible. Similarly, for the Oswestry Pain Questionnaire, the scores were expressed as a percentage of the maximum score possible.

### Results

The mean reduction in the pain questionnaire score following a single pamidronate treatment was 25% [standard error (s.e.) 3%, range 0-75%]. Twenty patients (59%) showed a >20% improvement and were classified as responders (Table III). Figure 1 shows the pain score following treatment expressed as a percentage of baseline (mean values ± s.e.). The median duration of symptomatic response was 12 (range 4-24+) weeks. None of the responding patients received palliative radiotherapy to any site during this symptomatic response. However, one patient classed as a responder commenced pamidronate within 2 weeks of a course of radiotherapy to spinal metastases, and the latter may have contributed to the response observed.

Table IV shows changes in the assessment of quality of life and mobility. At 4 weeks a 12% reduction from baseline (absolute reduction 4.1%) in the mean overall QOL score was seen, attributable to improvements in the physical and psychological domains rather than functional. Those patients showing a subjective response to treatment, as defined above, recorded a significant reduction in RSCL score (improvement in QOL) from 35% (s.e. 3.5%) before treatment to 29% (s.e. 3.1%) at 4 weeks. No significant improvement was observed in the non-responders. The Oswestry Questionnaire also showed a reduction in the mean percentage score, from 52% (s.e. 3.1%) to 43% (s.e. 3.0%) indicating a reduction in the impact of pain on normal social function and activities of daily living ($P = < 0.01$).

Treatment was well tolerated with minor symptoms occurring only after the first treatment. Eight patients (24%) experienced fever and transient rigors after the first infusion but this did not occur on subsequent treatments. One patient experienced diarrhoea and another nausea and vomiting. Four patients had biochemical evidence of hypocalcaemia

**Figure 1** Changes in pain score. Mean value and standard error (s.e.) are shown. Pain score calculated by combining the individual scores for pain, analgesic consumption and WHO performance status.

**Figure 2** Changes in urinary calcium excretion expressed as a molar ratio of urinary calcium to urinary creatinine. Mean value and standard error (s.e.) are shown.

**Table IV** Serial values for the Rotterdam Symptom Checklist (RSCL) Quality of Life Assessments and the Oswestry Mobility Questionnaires. Values are mean percentages of maximum possible scores

| | *Week on study* | | | | | | |
|---|---|---|---|---|---|---|---|
| | *0* | *1* | *2* | *3* | *4* | *6* | *8* |
| RSCL | | | | | | | |
| All patients | 34 | 34 | 33 | 32 | 32 | 28 | 23 |
| Psychological domain | 30 | 29 | 28 | 28 | 24 | 23 | 23 |
| Physical domain | 30 | 28 | 25 | 25 | 24 | 22 | 22 |
| Functional domain | 48 | 48 | 46 | 49 | 48 | 42 | 41 |
| All domains | 36 | 35 | 33 | 34 | 32[b] | 29[b] | 29[b] |
| Responders | 20 | 20 | 20 | 20 | 20 | 20 | 15 |
| All domains | 35 | 34 | 31 | 32 | 29 | 27[b] | 26[b] |
| Non-responders | 14 | 14 | 13 | 12 | 12 | 8 | 8 |
| All domains | 38 | 36 | 34 | 36 | 35 | 33 | 34 |
| Oswestry score | 34 | 34 | 33 | 32 | 32 | 28 | 23 |
| | 52 | 44[a] | 44[a] | 44[a] | 43[a] | 41[a] | 38[a] |

[a]$P = <0.01$ *vs* baseline. [b]$P = <0.05$ *vs* baseline.

(adjusted calcium 1.89–2.01 mmol $l^{-1}$). No patient experienced symptomatic hypocalcaemia. As expected, urinary calcium excretion fell significantly following treatment (Figure 2) and remained below the baseline value for 4–24 + weeks (median 12 weeks).

Theoretically, rapid infusion of large doses of pamidronate could lead to hypocalcaemia. In five of the patients receiving pamidronate over 2 h, we measured the total and non-ionised levels of calcium before and immediately after the infusion of pamidronate. The mean total serum calcium was 2.28 [standard deviation (s.d.) of 0.14] mmol $l^{-1}$ before treatment and 2.21 (s.d. 0.10) mmol $l^{-1}$ after the infusion, and the ionised serum calcium 1.22 (s.d. 0.04) mmol $l^{-1}$ and 1.15 (s.d. 0.08) mmol $l^{-1}$ before and after treatment respectively.

To date, 21 patients have been retreated. Eight out of 15 responders to their initial treatment showed a second reduction in pain score of >20%, but this was not seen in any of the six non-responders. Five patients have remained well with maintained symptomatic response for more than a year with no additional treatment for their disease other than repeat infusions of pamidronate every 3–6 months.

### Discussion

The bisphosphonates are an important advance in the treatment of metastatic bone disease, producing symptomatic relief (Coleman *et al.*, 1988; Morton *et al.*, 1988; Burckhardt *et al.*, 1989; Grabelsky *et al.*, 1991; Ernst *et al.*, 1992; Tyrrell *et al.*, 1993) and preventing skeletal morbidity (Paterson *et al.*, 1993; van Holton-Verzantvoort *et al.*, 1993). For pain relief parenteral treatment has proved most effective, with oral therapy limited somewhat by problems of poor absorption and gastrointestinal toxicity. To date, this has required regular outpatient visits to receive infusions of bisphosphonate, and in the studies mentioned above intravenous pamidronate was given every 2–4 weeks as the only systemic treatment for patients with metastatic bone disease. All of these studies reported similar results, with subjective benefit occurring in about one half of patients.

It is well recognised that the majority of an intravenous infusion of pamidronate is adsorbed onto the bone surface (Daley Yates *et al.*, 1991) where it remains bound to hydroxyapatite for a very long time – probably years. Therefore, a single infusion can theoretically have a prolonged duration of action on the osteoclast, causing sustained inhibition of bone resorption. Indeed this is seen following treatment of Paget's disease of bone (Fitton & McTavish, 1991). Our study has shown a high frequency of symptomatic response, achieved following a single infusion of a relatively high dose of pamidronate. Fifty-nine per cent of patients reported useful benefit lasting several weeks, and this could be reinduced by repeat treatments. The improvements in quality of life, as measured by the RSCL, and reduction in the social and functional impact of pain, shown by the Oswestry Questionnaire, provide additional evidence for the beneficial effects of treatment.

Our results support a small pilot study from South Africa where patients received a single infusion of 90 mg of pamidronate, again with useful subjective response (Hacking *et al.*,

1991). Previous studies have also reported healing of lytic metastases in approximaely one-quarter of patients (Coleman & Purohit, 1993; Tyrell *et al.*, 1993). In our present study however, the patients had sclerotic or mixed lytic/sclerotic metastases, making radiographic assessment unreliable, so we are unable to comment on the structural effects of single doses.

Retreatment of these patients was originally intended to be 'on demand' for those showing subjective benefit. However, six patients who failed to meet our criteria for response also received a second treatment. Interestingly, a second response was seen only in those showing symptomatic response to the first treatment. The bisphosphonates are expensive drugs and, although confirmatory studies are needed, our data suggest that the most cost-efficient use of bisphosphonates is to limit repeated treatment to those who have shown a clear subjective response to the first treatment.

The ability to be able to achieve symptomatic response in sclerotic metastatic bone disease through selective inhibition of bone resorption is an important clinical observation and confirms previous small studies with pamidronate (Clarke *et al.*, 1991) and clodronate (Adami & Mian, 1989) in prostate cancer. This effect is possible because there is always an important lytic component present (even in patients with predominantly sclerotic metastases on plain radiographs) in which the osteolysis can only be demonstrated either histologically or in some instances, by computerised tomographic (CT) scanning (Galasko, 1976; Coleman, 1991). The sclerotic radiographic appearances mask the uncoupling and imbalance of bone remodelling that occurs in many patients with bone metastases, with the excessive new bone formation occurring away from sites of osteoclastic bone resorption. Most probably it is the lytic component that causes metastatic bone pain, as pain is not a major feature of conditions such as osteopetrosis or myelofibrosis in which pure bone sclerosis occurs.

As in previous studies, it is unclear why only some patients respond to pamidronate. Inhibition of bone resorption, as measured by urinary calcium excretion, occurs in $>95\%$ of patients after 120 mg of pamidronate, and most also show a reduction in other markers of bone resorption such as collagen pyridinium cross-links and hydroxyproline excretion (Purohit *et al.*, in press). No relationship between symptomatic and biochemical response could be determined. Subjective response appeared to be unrelated to tumour type, the type of bone metastases as evident on radiological examination (i.e. lytic, sclerotic and/or mixed lytic and sclerotic) or amount or type of previous systemic treatment. The pretreatment pain score was slightly lower in responding patients than non-responders (8.7 *vs* 10.9), but this difference did not reach statistical significance.

The maximum safe dose of pamidronate has never been defined. In the only published phase I study of pamidronate, Body *et al.* (1987) assessed six different dose levels for the treatment of hypercalcaemia of malignancy. Treatment was well tolerated except for one obese patient who received a total dose of 285 mg of pamidronate ($3\ mg\ kg^{-1}$) infused over 2 h, and in whom fever and hypotension were reported. In our series, treatment was safe, even when given over 2 h, and well tolerated. Although all our patients were normocalcaemic at the beginning of the pamidronate infusion, significant hypocalcaemia was not a problem. Four out of 34 patients showed biochemical evidence of hypocalcaemia, but this was of no clinical significance and reverted to normal in 3–4 weeks. There may be scope for further dose escalation in the future. Whether this could lead to a greater or more sustained response to treatment is not known but, in view of the very long half-life of bisphosphonates in bone, is worthy of future study with a view to reducing hospital visits for palliative therapy to a minimum.

There was no evidence of renal toxicity with either the 12 or the 2 h infusions of pamidronate, and in particular the three patients with renal impairment before treatment (123, 172 and 238 $\mu mol\ l^{-1}$ respectively) tolerated treatment equally well.

This study adds further to the increasing number of publications identifying the bisphosphonates as a useful addition to the range of palliative treatments for metastatic bone disease. There remains the possibility of a placebo response contributing to our results, and having shown that this treatment is feasible and well tolerated we are now conducting a randomised placebo-controlled study. Nevertheless, for the majority of this cohort of heavily pretreated patients, outpatient treatment with intravenous high-dose pamidronate was a valuable new treatment approach.

### References

ADAMI, S. & MIAN, M. (1989). Clodronate therapy of metastatic bone disease in patients with prostatic cancer. In *Bisphosphonates and Tumour Osteolysis*, Brunner, K.W., Fleisch, H. & Senn, H.-J. (eds) pp. 67–72. Springer: Heidelberg.

BODY, J.J., POT, M., BORKWSKI, A., SCULIER, J.P. & KLASTERSKY, J. (1987). Dose response study of aminohydroxypropylidene bisphosphonate in tumor-associated hypercalcemia. *Am. J. Med.*, **82,** 957.

BURCKHARDT, P., THIEBAUD, D., PEREY, L. & VON FLIEDNER, V. (1989). Treatment of tumour induced osteolysis by APD. *Rec. Res. Cancer Res.*, **116,** 54.

CLARKE, N.W., HOLBROOK, I.B., MCCLURE, J. & GEORGE, N.J. (1991). Osteoclast inhibition by pamidronate in metastatic prostate cancer: a preliminary study. *Br. J. Cancer*, **63,** 420–423.

COLEMAN, R.E. & RUBENS, R.D. (1987). The clinical course of bone metastases from breast cancer. *Br. J. Cancer*, **55,** 61.

COLEMAN, R.E. & PUROHIT, O.P. (1993). Osteoclast inhibition for the treatment of bone metastases. *Cancer Treat. Rev.*, **19,** 79.

COLEMAN, R.E. (1991). Assessment of response to treatment. In *Bone Metastases – Diagnosis and Treatment*, Rubens, R.D. & Fogleman, I. (eds) p. 99. Springer: London.

COLEMAN, R.E., WOLL, P.J., MILES, M., SCRIVENER, W. & RUBENS, R.D. (1988). 3-Amino-1,1 hydroxypropylidene bisphosphonate (APD) for the treatment of bone metastases from breast cancer. *Br. J. Cancer*, **58,** 621.

DALEY-YATES, P.T., DODWELL, D.J., PONGCHAIDECHA, M., COLEMAN, R.E. & HOWELL, A. (1991). The clearance and bioavailability of pamidronate in patients with breast cancer and bone metastases. *Calcif. Tissue Int.*, **49,** 433.

DE HAES, J.C.M., KNIPPENBERG, F.C.E. & NEIJT, J.P. (1990). Measuring psychological and physical distress in cancer patients: application of the Rotterdam Symptom Checklist. *Br. J. Cancer*, **62,** 1034.

ERNST, D.S., MACDONALD, N., PATERSON, A.H.G., JENSON, J., BRASHER, P. & BRUERA, E. (1992). A double blind, crossover trial of intravenous clodronate in metastatic bone pain. *J. Pain Symptom Management*, **7,** 4.

FAIRBANK, J.C.T., COUPER, J., DAVIES, J.B. & O'BRIEN, J.P. (1980). The Oswestry low back questionnaire. *Physiotherapy*, **66,** 271.

FITTON, A. & MCTAVISH, D. (1991). Pamidronate – a review of its pharmacological properties and therapeutic efficacy in resorptive bone disease. *Drugs*, **41,** 289.

GALASKO, C.B.E. (1976). Mechanisms of bone destruction in the development of skeletal metastases. *Nature*, **263,** 507.

GRABELSKY, S., LIPTON, A., HARVEY, H., GLOVER, D., BROWNING, S., DILLMAN, R., FRAM, R.J., GEORGE, S., KELLER, A., MILLER, A. & SEAMAN, J. (1991). Pamidronate disodium (APD) – a dose-seeking study in patients with breast cancer. *Proc. Am. Soc. Clin. Oncol.*, **10,** 42 (abstract 41).

HACKING, A., GUDGEON, C.A., MCNAUGHTON, D. & DENT, D.M. (1991). Disodium pamidronate (Aredia) as single infusion monotherapy in the treatment of bone metastases from breast cancer. In *Osteoclast Inhibition in the Management of Malignancy Related Bone Disorders*, Bijvoet, O.L.M. & Lipton, A. (eds) pp. 45–53. Hogrefe & Huber: Bern.

MORTON, A.R., CANTRILL, J.A., PILLAI, G.V., MCMAHON, A., ANDERSON, D.C. & HOWELL, A. (1988). Sclerosis of lytic bone metastases after disodium aminohydroxypropylidene bisphosphonate (APD) in patients with breast cancer. *Br. Med. J.*, **297**, 772.

PATERSON, A.H.G., POWLES, T.J., KANIS, J.A., MCCLOSKEY, E., HANSON, J. & ASHLEY, S. (1993). Double blind controlled trial of clodronate in patients with bone metastases from breast cancer. *J. Clin. Oncol.*, **11**, 59.

PUROHIT, O.P., DICKSON, I. & COLEMAN, R.E. (1994). Effect of a single high dose treatment with pamidronate on markers of bone resorption in patients with bone metastases. *Br. J. Cancer*, **69** (Suppl. XXI), 45.

RALSTON, S.H., GARDNER, M.D., DRYBURGH, F.J., JENKINS, A.S., COWAN, R.A. & BOYLE, I.T. (1985). Comparison of aminohydroxypropylidene diphosphonate, mithramycin, and corticosteroids/calcitonin in treatment of cancer-associated hypercalcaemia. *Lancet*, **i**, 907.

TYRRELL, C.J., BRUNING, P.F., MAY-LEVIN, F., ROSE, C. & FORD, J.M. (1993). Non comparative multi-centre open trial in patients with breast cancer and bone metastases to investigate the effect of apd (pamidronate) on bone healing, morbidity and bone metabolism. *Breast*, **2**, 202.

TYRELL, C.J., MADSEN, E.L., COLLINSON, M., FORD, J.M. & COLEMAN, T. (1993). A study of renal function of four infusions of pamidronate 90 mg given over 60 min one week apart. *Eur. J. Cancer*, **29A** (Suppl. 6), 282.

VAN HOLTEN-VERZANTVOORT, A.T., KROON, H.M., BIJVOET, CLETON, F.J., BEEX, L.V.A.M., BLIJHAM, G., HERMANS, J., NEIJT, J.P., PAPAPOULOS, S.E., SLEEBOOM, H.P., VERMEY, P. & ZWINDERMAN, A.H. (1993). Palliative bone treatment in patients with bone metastases from breast cancer. *J. Clin. Oncol.*, **11**, 491–498.

WHO (1979). *Handbook for Reporting Results of Treatment*. WHO: Geneva.